# Optimization of Light Intensity, Temperature, and Nutrients to Enhance the Bioactive Content of Hyperforin and Rutin in St. John’s Wort

**DOI:** 10.3390/molecules25184256

**Published:** 2020-09-16

**Authors:** Chia-Hung Kuo, Yi-Chin Chou, Kuo-Chun Liao, Chwen-Jen Shieh, Tzu-Shing Deng

**Affiliations:** 1 Department of Seafood Science, National Kaohsiung University of Science and Technology, Kaohsiung 811, Taiwan; kuoch@nkust.edu.tw; 2 Department of Agronomy, National Chung Hsing University, Taichung 402, Taiwan; hoyoo1210@gmail.com (Y.-C.C.); zcbm0307@gmail.com (K.-C.L.); 3 Biotechnology Center, National Chung Hsing University, Taichung 402, Taiwan

**Keywords:** St. John’s wort, leaf extracts, hyperforin, rutin, melatonin, optimization, RSM

## Abstract

St. John’s wort (*Hypericum perforatum* L.) is a medicinal plant that alleviates depression and other disorders due to its abundance of active ingredients. Hyperforin, rutin, and melatonin are the main active, and important, ingredients in St. John’s wort that alleviate depression. In order to investigate the optimal conditions for accumulating these active ingredients, design of experiments and response surface methodology (RSM) was employed in this study. Two-month-old St John’s wort plants were cultivated in growth chambers at varying temperatures, light intensities, and nutrient solution concentrations before analysis by HPLC, for determining differences in hyperforin, rutin, and melatonin content. The results showed that hyperforin and rutin contents were significantly influenced by temperature (18–23 °C) and light intensity (49–147 μmol m^−2^ s^−1^ photosynthetic photon flux density (PPFD)), whereas Hoagland’s nutrient solution concentration (25–75%) had little effect. The accumulation of melatonin might not be influenced by cultivation conditions. Light intensity and temperature are easily controlled environmental factors in artificial cultivation, both of which are related to secondary metabolite production in the plant. Based on RSM, the optimal conditions for the accumulation of hyperforin and rutin were obtained. The maximum content of hyperforin was 5.6 mg/g, obtained at a temperature of 19 °C, a nutrient solution concentration of 45%, and a light intensity of 49 μmol m^−2^ s^−1^ PPFD. The maximum content of rutin was 3.8 mg/g obtained at a temperature of 18 °C, a nutrient solution concentration of 50%, and a light intensity of 147 μmol m^−2^ s^−1^ PPFD. This evaluation of suitable conditions for the accumulation of bioactive compounds in St. John’s wort can be applied to plant factories on a large scale.

## 1. Introduction

St. John’s wort (*Hypericum perforatum* L.), belonging to the Hypericaceae family, is a perennial herb native to Europe, Western Asia, and North Africa that has been naturalized to North America, South America, and Australia [1,2]. St. John’s wort has been used throughout history as a medicinal herb to treat several diseases, including colds, chest congestion, headaches, fever, snake bites, asthma, tuberculosis, skin problems, wounds, bruises, and burns [3,4]. Several clinical studies have demonstrated the effectiveness of St. John’s wort extract in the treatment of depression [5,6]. St. John’s wort extract has also been suggested for acquired immunodeficiency syndrome (AIDS) and cancer treatments [7]. The major active constituents of St. John’s wort are hyperforin, rutin, and melatonin [8,9]. Hyperforin is believed to be responsible for antidepressant activity [10], but also exhibits anti-inflammatory [11], antibacterial, and antitumoral effects [12]. Rutin has been shown to be an effective antioxidant, and demonstrates potential anti-inflammatory and cardioprotective activities [13,14], while melatonin is an effective free radical scavenger and antioxidant [15,16]. In addition, melatonin exhibits anticancer properties and can prevent age-related cardiovascular disorders [17]. The German Commission E designated St. John’s wort as an approved herb in 1984, and it is currently one of the most widely consumed medicinal plants in the world [1,18]. The importance of St. John’s wort as a dietary supplement has significantly increased in the last few years. The annual market for St. John’s wort has reached $210 million in the United States alone and over $570 million worldwide [19].

St. John’s worts grown in different regions have varying concentrations of bioactive compounds [20]. Environmental factors such as temperature, light quality, light intensity [21,22], and nutrient availability affect the levels of secondary metabolites in plant tissues, resulting in varied chemical profiles [23,24]. As the phytochemical composition of St. John’s wort is dependent on season, region, and temperature, the quality of plants grown in the field is difficult to control [2]. For industrial production, raw materials with less variation in composition are preferred. The plant factory can provide the required environment according to the plant, and all kinds of natural plants can be cultivated [25,26]. However, the plant factory is a high-capital and high-intensiveness production method. The plant factory is mainly used for high value-added products, such as functional plants for health care and beauty (such as increasing the content of vitamins, lycopene, anthocyanins, antioxidant power, etc.) and herbal medicine. Although the plant factory provides complete environmental control, it is still necessary to know exactly what kind of environmental conditions the cultivated plants need at different growth stages, to set the relevant environmental parameters. As such, determining the optimal temperature and light intensity for chemical accumulation, as well as plant growth and development, is integral to obtaining a consistent composition and increased concentration of phytochemicals.

Response surface methodology (RSM) is a collection of mathematical and statistical techniques for designing experiments, building models, evaluating the relative significance of multiple independent variables, and determining the optimal conditions for the desired response [27,28,29,30]. It is used to evaluate the effects of various parameters and their interactions via a small number of experiments. The Box-Behnken experimental design is rotatable and can reduce the number of experimental runs, when evaluating the polynomial model, since the distance between any experimental point to the center point is equidistant. It is a very efficient experimental design applied to RSM [31,32,33,34]. Compared to a one-factor-at-a-time design, which is adopted most frequently in the literature, the experimental design and RSM were more efficient in reducing the number of experimental runs and the time needed to investigate optimal conditions. However, little information is available on the relationship between environmental factors and the level of secondary metabolites in St. John’s wort. Similarly, literature regarding the use of RSM in optimizing the production of secondary metabolites is also limited.

The total concentration of hyperforin, rutin, and melatonin in St. John’s wort extract determines the quality of the phytomedicine. In this study, RSM was used to investigate the relationship between environmental factors (temperature, light intensity, and nutrient solution concentration) and the accumulation of secondary metabolites (hyperforin, rutin, and melatonin) in the plant. In order to increase the level of desired phytochemicals, the optimal conditions for production of the three bioactive compounds were evaluated.

## 2. Results and Discussion

### 2.1. High-Performance Liquid Chromatography Traces of St. John’s Wort Leaf Extracts

**Figure 1:**
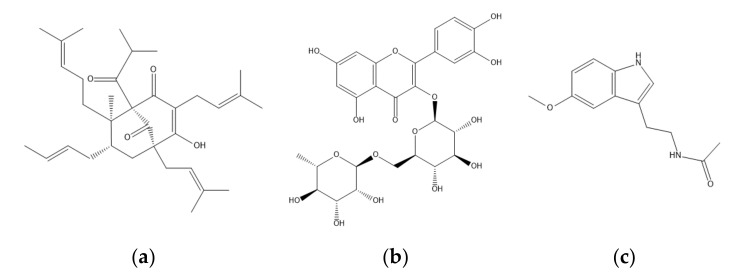
Chemical structures of the bioactive constituents of St. John’s wort: (**a**) hyperforin, (**b**) rutin, and (**c**) melatonin.

**Figure 2:**
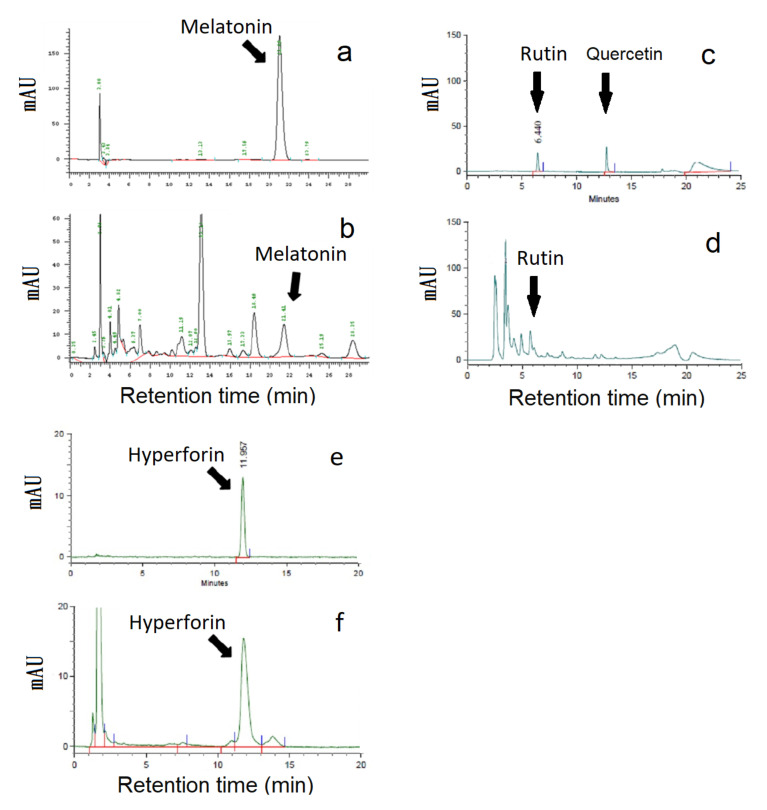
Representative HPLC chromatograms of standard (**a**) melatonin, (**c**) rutin, and (**e**) hyperforin and of St. John’s wort leaf extracts (**b**) melatonin, (**d**) rutin, and (**f**) hyperforin. The arrows indicate the peaks of target compounds in the HPLC chromatogram.

The St. John’s wort leaf extracts were analyzed by HPLC methods. The major active constituents in leaf extracts were hyperforin, rutin, and melatonin, as shown in Figure 1. Since the extraction methods for melatonin, rutin, and hyperforin were different, three HPLC analytical methods were used for measuring three target components. A representative HPLC result is shown in Figure 2. The retention times of melatonin, rutin, and hyperforin were 21.41, 6.44, and 11.96 min, respectively. The HPLC chromatograms of the leaf extracts showed the three target compounds had the same retention times as the peaks from standards of melatonin, rutin, and hyperforin. A representative HPLC result is shown in Figure 2. The results indicated that the HPLC method could be utilized for analyzing the accumulation of secondary metabolites (hyperforin, rutin, and melatonin) in the leaves of St. John’s wort under various cultivation conditions.

### 2.2. Statistical Model Fitting

The main objective of this work was to develop and evaluate a statistical approach to better understand the relationship between the three independent variables (cultivation temperature, light intensity, and nutrient solution concentration) and the accumulation of bioactive compounds in the leaves of St. John’s wort. A multiple RSM approach combined with a three-level, three-factor Box-Behnken design was employed in this study. The experimental design of the three independent variables’ effect on the response functions are listed in Table 1. The plants were cultivated under the design conditions. The leaves were harvested after two months for target component analysis and the results are summarized in Table 1. Among the various cultivation conditions, the highest contents of hyperforin and rutin were obtained from run no. 1 (temperature 18 °C; light intensity 49 μmol m^−2^ s^−1^ PPFD; nutrient 50%) and run no. 4 (temperature 18 °C; light intensity 147 μmol m^−2^ s^−1^ PPFD; nutrient 50%), respectively. The lowest contents of hyperforin and rutin were obtained from run no. 13 (temperature 28 °C; light intensity 98 μmol m^−2^ s^−1^ PPFD; nutrient 25%) and run no. 6 (temperature 23 °C; light intensity 49 μmol m^−2^ s^−1^ PPFD; nutrient 75%), respectively. However, the amount of melatonin varied from 0.05 to 0.07 μg/g indicated the cultivation conditions had less influence.

The manipulated and response variables were analyzed to fit a regression equation (model) that could predict the response within a given range of independent variables. The response surface regression (RSREG) procedure from SAS was employed to fit the second order polynomial equation to the experimental data. Second-order polynomial equations were given as below for contents of hyperforin (Equation (1)), rutin (Equation (2)), and melatonin (Equation (3)).

Hyperforin content (Y_1_):

Y_1_ = −3.519320 + 0.815830X_1_ − 0.076255X_2_ + 0.152883X_3_ − 0.027130X_1_^2^ + 0.003274X_1_X_2_ − 0.000038942X_2_^2^ + 0.001554X_1_X_3_ − 0.000041224X_2_X_3_ − 0.001926X_3_^2^ (1)

Rutin content (Y_2_):

Y_2_ = 2.518185 − 0.652315X_1_ + 0.096335X_2_ + 0.052694X_3_ + 0.017890X_1_^2^ − 0.001584X_1_X_2_ − 0.000180X_2_^2^ − 0.000138X_1_X_3_ − 0.000023878X_2_X_3_ − 0.000456X_3_^2^ (2)

Melatonin content (Y_3_):

Y_3_ = 0.029940 + 0.004965X_1_ − 0.000333X_2_ − 0.001332X_3_ − 0.000065X_1_^2^ − 0.000007143X_1_X_2_ + 0.000001822X_2_^2^ − 0.000016X_1_X_3_ + 0.000004082X_2_X_3_ + 0.0000138X_3_^2^ (3)

where Y is the predicted content, X_1_ is the temperature, X_2_ is the light intensity, and X_3_ is the nutrient solution concentration.

**Figure 3:**
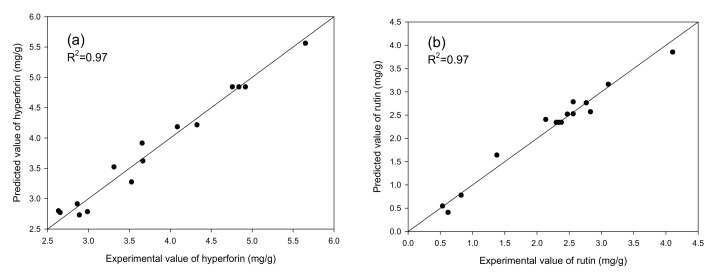
The relationship between predicted and experimental contents of (**a**) hyperforin and (**b**) rutin.

An analysis of variance (ANOVA) was performed on the model once it was fitted to the experimental data. ANOVA data from Table 2 also indicated that the second-order polynomial models (Equations (1) and (2)) for contents of hyperforin (Y_1_) and rutin (Y_2_) were statistically significant and adequately represented the actual relationship between the responses and the variables, with a small model *p*-value (*p* < 0.05) and satisfactory coefficient of determination (R^2^ = 0.97). However, the coefficient of determination for the melatonin model was only 0.76, with a *p*-value of 0.2751, indicating it was not statistically significant. This suggests that the accumulation of melatonin might not be influenced by cultivation temperature, light intensity, or nutrient solution concentration. Melatonin concentration in plants is typically affected by genotype, environmental factors, and stage of development. As such, the concentration of melatonin can vary among different cultivars of the same species [35]. In a recent study it was reported that seed germination is associated with marked increases in melatonin concentration (2–3 fold) and that the germinated seeds of edible species may have potential as a food that raises melatonin levels in plasma [36]. Melatonin acts as a biostimulator in situations of abiotic stress, regulating key elements expressed against environmental stressors, such as extreme heat, cold, drought, salinity, alkalinity, heavy metals (including boron), chemical agents (herbicides, toxics), and high solar and UV radiation [37,38,39]. This could be the reason that temperature, light intensity, and nutrient solution concentration had less influence on melatonin yield. In contrast, the plots of experimental contents (%) versus those calculated from Equations (1) and (2) indicated a good fit for hyperforin and rutin, as seen in Figure 3. The models for hyperforin and rutin were therefore used to construct three-dimensional response surface plots to predict the relationship between independent and dependent variables. The overall effect of the three manipulated variables was also analyzed by the joint test. The results (Table 3) revealed that temperature (X_1_), light intensity (X_2_), and nutrient solution concentration (X_3_) were important factors in hyperforin content. However, only light intensity (X_2_) had a significant effect (*p* > 0.05) on rutin content.

### 2.3. Hyperforin Content

**Figure 4:**
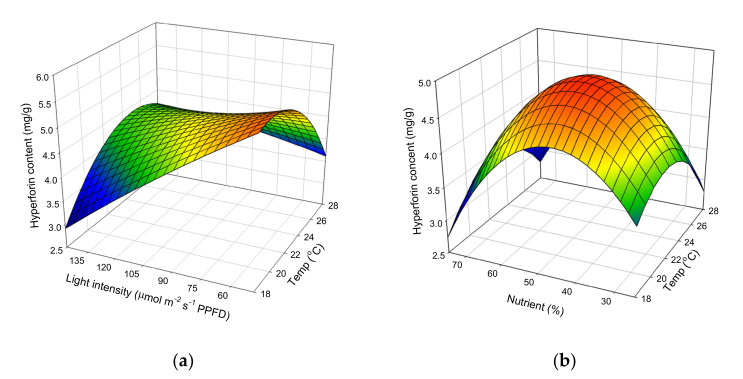
Response surface plot showing the effects of cultivation (**a**) temperature, light intensity, and their mutual interaction on the hyperforin content. (**b**) temperature, nutrient concentration, and their mutual interaction on the hyperforin content.

The effect of cultivation temperature and light intensity on hyperforin content, when nutrient concentration is kept at a constant 50%, is shown in Figure 4a. At the lowest temperature and light intensity, hyperforin content was 5.56 mg/g, but when light intensity was increased to 147 μmol m^−2^ s^−1^ PPFD and the temperature raised to 18 °C, the hyperforin content decreased to 2.92 mg/g. Figure 4b shows the effects of cultivation temperature, nutrient solution concentration, and their mutual interaction on hyperforin content when light intensity is kept at a constant 98 μmol m^−2^ s^−1^ PPFD. Increases in nutrient concentration (from 25% to 50%) and temperature (from 18 °C to 23 °C) enhanced hyperforin content. However, once nutrient concentration and temperature, respectively, increased above 50% and 23 °C, there was a gradual decline in the response. Brechner et al. reported that in controlled environments, hyperforin concentration in St John’s wort was consistently higher at a light intensity of 90 μmol m^−2^ s^−1^ PPFD, versus light intensities of 160 or 340 μmol m^−2^ s^−1^ PPFD [40]. Temperature is an important abiotic factor that greatly influences the growth, yield, and quality of crop plants, and the morphology of seedlings. Liu et al. reported that extreme high and low night temperatures had a negative effect on primary and secondary metabolites, while a mild night temperature significantly increased the concentration of soluble sugars, starches, total phenols, and flavonoids in *Astragalus membranaceus* and *Codonopsis lanceolata* [41]. Couceiro et al. investigated the effect of two different temperatures and found that hyperforin concentration remained higher at 25 °C than at 30 °C [20]. These results agree with our own, demonstrating the importance of controlling the environment in order to produce major bioactive compounds in St. John’s wort.

### 2.4. Rutin Content

**Figure 5:**
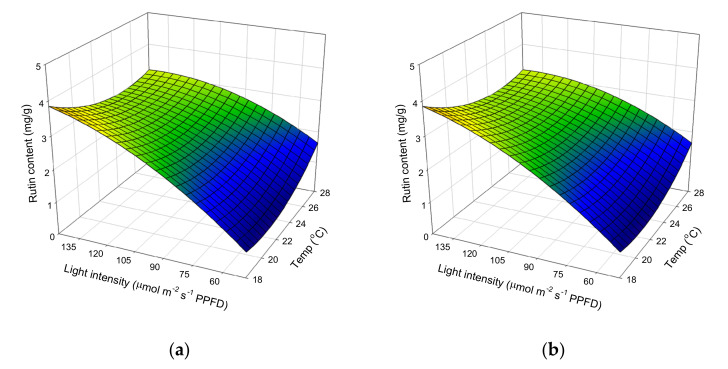
Response surface plot showing the effects of cultivation (**a**) temperature, light intensity, and their mutual interaction on the rutin content; (**b**) light intensity, nutrients, and their mutual interaction on the rutin content.

The effect of cultivation temperature and light intensity on rutin content, when nutrient solution concentration was kept at a constant 50%, is shown in Figure 5a. At any given temperature, increasing light intensity resulted in higher rutin content. At the lowest temperature (18 °C) and highest light intensity (147 μmol m^−2^ s^−1^ PPFD), the maximum rutin content was reached (3.84 mg/g). However, at light intensities of 49–98 μmol m^−2^ s^−1^ PPFD and 98–147 μmol m^−2^ s^−1^ PPFD, increasing cultivation temperature resulted in only slight increases and slight decreases in rutin content, indicating that temperature had little influence. The effect of light intensity and nutrient solution concentration on rutin content, when temperature was kept at a constant 23 °C, is shown in Figure 5b. At any given nutrient solution concentration, increasing light intensity from 49 to 147 μmol m^−2^ s^−1^ PPFD increased rutin content from 0.5 to 3 mg/g, indicating the importance of light intensity on rutin content; conversely, nutrient solution concentration had an insignificant effect. Temperature and light are the major environmental factors affecting plant physiology, especially photosynthesis and plant development. Increases in secondary metabolite production in plants are attributed to changes in the photosynthetic rate [36]. According to our results, light intensity has a greater influence than temperature, as it triggered the use of available carbon in the plant tissues, for the biosynthesis of rutin.

### 2.5. Attaining Optimal Cultivation Conditions for the Contents of Hyperforin and Rutin

**Figure 6:**
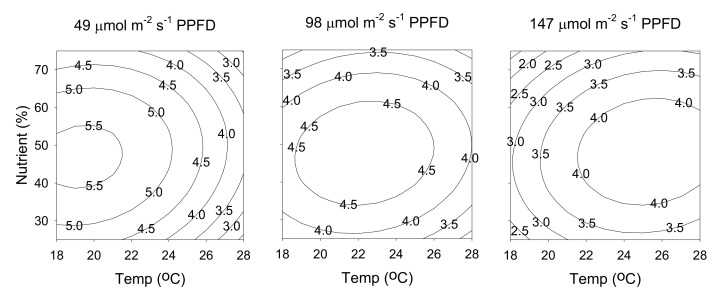
Contour plots of hyperforin content. The numbers inside the contour plots indicate content (mg/g) at given cultivated conditions.

**Figure 7:**
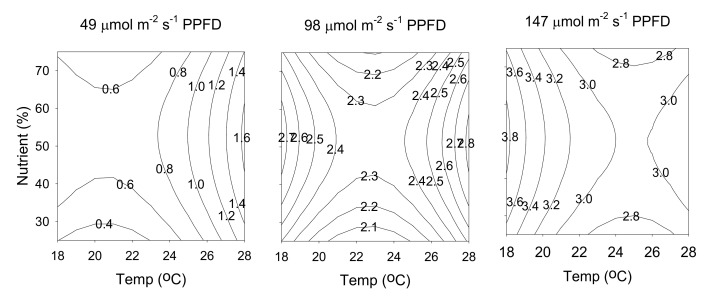
Contour plots of rutin content. The numbers inside the contour plots indicate content (mg/g) at given cultivated conditions.

The relationship between cultivation parameters and hyperforin content can be better understood by examining the series of contour plots (Figure 6) generated from the predicted model (Equation (1)), by holding constant at a specific light intensity (49, 98, or 147 μmol m^−2^ s^−1^ PPFD). The three contour plots in Figure 6 exhibit similar behaviors, in which the content of hyperforin increased as light intensity decreased. The maximum value of 5.6 mg/g was obtained at a temperature of 19 °C, a nutrient solution concentration of 45%, and a light intensity of 49 μmol m^−2^ s^−1^ PPFD. The relationship between cultivation parameters and rutin content generated from the predicted model (Equation (2)), by holding constant at a specific light intensity (49, 98, or 147 μmol m^−2^ s^−1^ PPFD), is shown in Figure 7. At 49 μmol m^−2^ s^−1^ PPFD, increasing the temperature also increased the content of rutin. Conversely, when light intensity was increased to 147 μmol m^−2^ s^−1^ PPFD, rutin content increased as temperature decreased. The maximum value of 3.8 mg/g was obtained at a temperature of 18 °C, a nutrient solution concentration of 50%, and a light intensity of 147 μmol m^−2^ s^−1^ PPFD.

### 2.6. Attaining Optimal Production Yields of Hyperforin and Rutin

The relationship between cultivation parameters and the production yield of leaf biomass is also an important consideration. The leaves of each plant were collected and weighted after two months for analyzing the production yields of leaf biomass, hyperforin, and rutin; the results are summarized in Table 4.

The RSREG procedure from SAS was employed to fit the second order polynomial equation to the experimental data. Second-order polynomial equations were given as below for production yields of leaf biomass (Equation (4)), hyperforin (Equation (5)), and rutin (Equation (6)).

Total leaves weight (Y_4_):

Y_4_ = −1.277170 + 0.132705X_1_ + 0.005424X_2_ + 0.004927X_3_ − 0.003855X_1_^2^ + 0.000161X_1_X_2_ − 0.000060027X_2_^2^ + 0.000886X_1_X_3_ + 0.000075510X_2_X_3_ − 0.000247X_3_^2^ (4)

Hyperforin yield (Y_5_):

Y_5_ = −15.742387 + 1.364381X_1_ − 0.011338X_2_ + 0.203744X_3_ − 0.039335X_1_^2^ + 0.003016X_1_X_2_ − 0.000345X_2_^2^ + 0.003710X_1_X_3_ + 0.000117X_2_X_3_ − 0.002816X_3_^2^ (5)

Rutin yield (Y_6_):

Y_6_ = −0.562464 − 0.219310X_1_ + 0.062954X_2_ + 0.018885X_3_ + 0.003702X_1_^2^ − 0.000206X_1_X_2_ − 0.000272X_2_^2^ + 0.002367X_1_X_3_ + 0.000303X_2_X_3_ − 0.000836X_3_^2^ (6)

where Y is the predicted product yield, X_1_ is the temperature, X_2_ is the light intensity, and X_3_ is the nutrient solution concentration.

An analysis of variance (ANOVA) was performed on the model once it was fitted to the experimental data. The analysis of variance (ANOVA) indicated that the second-order polynomial model (Equations (4)–(6)) was statistically significant and adequately represented the actual relationship between the responses and the variables, with a small *p*-value (<0.05) of the total model and a satisfactory coefficient of determination (R^2^ = 0.90, 0.96, and 0.99 for Equations (4)–(6), respectively).

**Figure 8:**
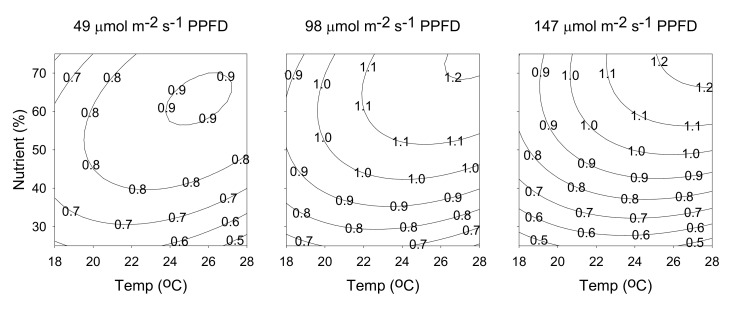
Contour plots of production yield of leaf biomass. The numbers inside the contour plots indicate production yield (g/plant) at given cultivated conditions.

The relationship between cultivation parameters and production yield of leaf biomass can be better understood by examining the series of contour plots (Figure 8) generated from the predicted model (Equation (4)), by holding constant at a specific light intensity (49, 98, or 147 μmol m^−2^ s^−1^ PPFD). The three contour plots in Figure 7 exhibit similar behaviors, in which the production yield of leaf biomass increased with temperature, light intensity, and nutrient solution concentration. The maximum value of 1.25 g/plant was obtained at a temperature of 28 °C, a nutrient solution concentration of 75%, and a light intensity of 147 μmol m^−2^ s^−1^ PPFD.

**Figure 9:**
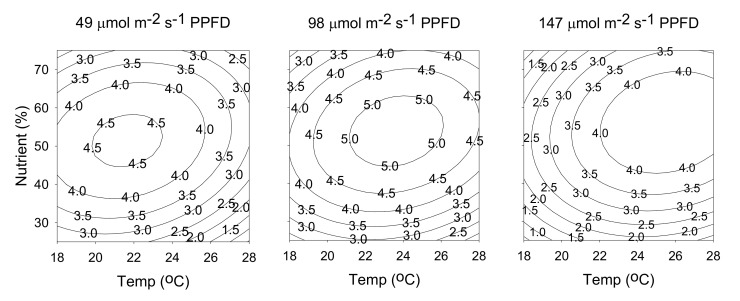
Contour plots of production yield of hyperforin. The numbers inside the contour plots indicate production yield (mg/plant) at given cultivated conditions.

**Figure 10:**
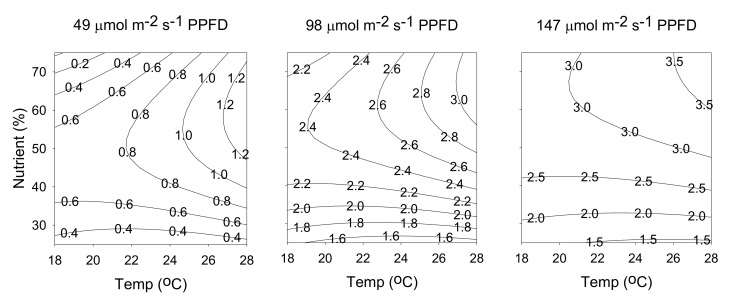
Contour plots of production yield of rutin. The numbers inside the contour plots indicate production yield (mg/plant) at given cultivated conditions.

The relationship between cultivation parameters and production yield of hyperforin generated from the predicted model (Equation (5)), by holding constant at a specific light intensity (49, 98, or 147 μmol m^−2^ s^−1^ PPFD), is shown in Figure 9. The maximum value of 5.19 mg/plant was obtained at a temperature of 23 °C, a nutrient solution concentration of 50%, and a light intensity of 98 μmol m^−2^ s^−1^ PPFD. The relationship between cultivation parameters and production yield of rutin generated from the predicted model (Equation (6)), by holding constant at a specific light intensity (49, 98, or 147 μmol m^−2^ s^−1^ PPFD). As shown in Figure 10, the three contour plots exhibit similar behaviors, the production yield of rutin increased with temperature, light intensity, and nutrient solution concentration. The maximum value of 3.76 mg/plant was obtained at a temperature of 28 °C, a nutrient solution concentration of 75%, and a light intensity of 147 μmol m^−2^ s^−1^ PPFD.

## 3. Materials and Methods

### 3.1. Materials

St. John’s wort seeds were obtained from the Institute of Botany, at the University of Jena in Germany. Hyperforin, rutin, and melatonin were purchased from Sigma-Aldrich (St. Louis, MO, USA). Unless otherwise noted, all reagents and chemicals were of analytical grade.

### 3.2. Growing Conditions

**Figure 11:**
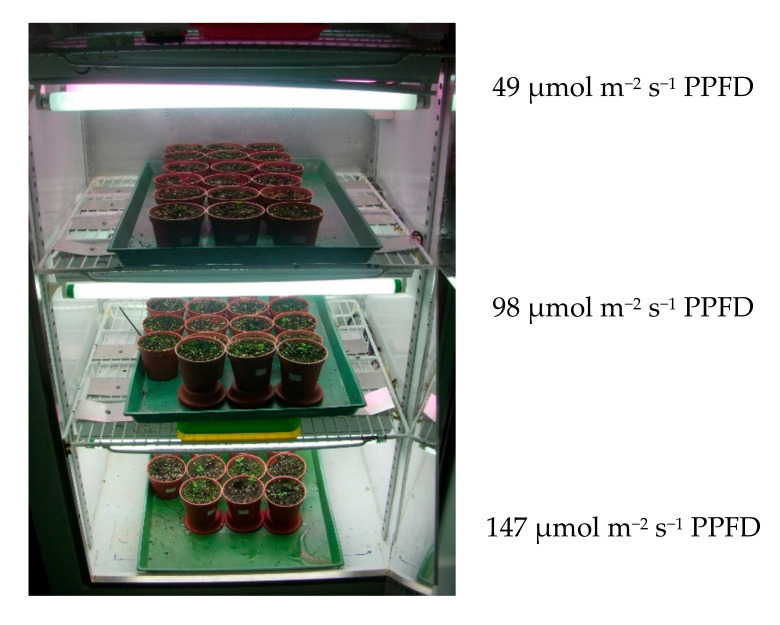
Growth conditions of St. John’s wort cultivated in growth chambers with light intensities of 49, 98, and 147 μmol m^−2^ s^−1^ PPFD, respectively.

St. John’s seeds were germinated at the same condition, then the similar seeds were placed on top of moist soil (peat, perlite and vermiculite 25:1:0.5, pH 5.6) in a 3 inch plastic flowerpot (9 × 6.2 × 6.8 cm^3^) after germination kept with 3 plants till harvest. Based on the required cultivation conditions, the photoperiod was set at 18 h light/6 h dark using growth chambers set at 18 °C, 23 °C, or 28 °C. Light intensity in the chambers was adjusted to 49, 98, and 147 μmol m^−2^ s^−1^ PPFD via fluorescent tubes (TL-D18W/865, PHILIPS) and plant lamps (FL-20BR18W, Xu Guang) enriched in blue light (400–500 nm) and red light (600–700 nm), as shown in Figure 11. Plants were watered daily with an appropriate amount of reverse osmosis water. 20 mL of Hoagland’s nutrient solution (25%, 50%, or 75% strength) was administered every two weeks after germination. Each experimental condition in Table 1 has three flowerpots. The first-third and two-thirds of the leaves at the top of the stem were taken for target components analysis after 2 months and stored in a −60 °C freezer for future analysis.

### 3.3. Extraction of Hyperforin, Rutin and Melatonin

Fresh St. John’s wort leaves were treated with liquid nitrogen and ground into powder.

Hyperforin was extracted according to the methods of Soelberg et al. [42]. Briefly, 0.25 g fresh leaf powder was added to a centrifuge tube with 2.5 mL of 80% aqueous methanol (containing 0.073 M (2-hydroxypropyl)-β-cyclodextrin adjusted to pH 2.5 with ortho-phosphoric acid). The centrifuge tube was placed in an ultrasonic bath for 10 min. After 10 min centrifugation at 5000 rpm, the supernatant was decanted and the extraction procedure repeated three times with the pellet. The collected supernatant was supplemented with 80% aqueous methanol to a final volume of 10 mL before HPLC analysis.

Rutin was extracted according to the methods of Ganzera et al. [43], with some minor modifications. Briefly, 0.25 g fresh leaf powder was added to 20 mL methanol in a 50 mL centrifuge tube. The tube was placed in an ultrasonic bath and extracted for 40 min. After 10 min centrifugation at 5000 rpm, the supernatant was used for HPLC analysis.

Melatonin was extracted according to the methods of Guerrero et al. [44]. Briefly, 0.3 g fresh leaf powder was added to a centrifuge tube with 10 mL sodium phosphate buffer (50 mM, pH 8.0) containing 5 μM butylated hydroxytoluene (BHT) as antioxidant. The tube was placed in an ultrasonic bath and extracted for 40 min followed by centrifugation at 5000 rpm for 10 min. The supernatant was purified twice by solvent partitioning using 12 mL ethyl acetate. The two organic phases (~12 mL each) were evaporated under vacuum at 40 °C. Dry residue was redissolved in 1 mL methanol for HPLC analysis.

### 3.4. Experimental Design and Statistical Analysis

A three-factor-three-level Box-Behnken experimental design consisting of 15 treatments was employed in this study. All treatments were implemented in a completely random order. The manipulated (independent) variables and selected levels were: cultivation temperature (18–28 °C), light intensity (49–147 μmol m^−2^ s^−1^ PPFD), and Hoagland’s nutrient solution concentration (25–75%). The responses were: hyperforin content, rutin content, and melatonin content in the leaves of the cultivated St. John’s wort; production yield of leaf biomass, production yield of hyperforin, and production yield of rutin for each St. John’s wort. Table 5 shows the independent factor (*Xi*) levels in the experimental design, in terms of coded and uncoded. The effect of the three independent variables (temperature, light intensity, and nutrient solution concentration) on the responses (*Yn*: hyperforin content, rutin content, melatonin content, production yield of leaf biomass, production yield of hyperforin, and production yield of rutin) was designed using polynomial response surface methodology. The second-order response function for the experiment was predicted using the following Equation:

(7) $$Y=\beta_{0}+\overset{3}{\underset{i=1}{\sum }}\beta_{i}x_{i}+\overset{3}{\underset{i=1}{\sum }}\beta_{ii}x_{i}^{2}+\overset{2}{\underset{i=1}{\sum }}\overset{3}{\underset{j=i+1}{\sum }}\beta_{ij}x_{i}x_{j}$$

where *Yn* is one of the six responses (hyperforin content, rutin content, melatonin content, production yield of leaf biomass, production yield of hyperforin, or production yield of rutin); *β_k_*_0_, *β_ki_*, *β_kii_*_,_ and *βk_ij_* are constant coefficients; and *Xi* and *Xj* are uncoded, independent variables. The fitness of the model was evaluated by the determination coefficient R^2^ and analysis of variance (ANOVA). The fitted polynomial equations were presented as surface and contour plots in order to understand the relationship between the responses and each factor’s experimental levels and to deduce the optimal conditions. Regression and analysis of variance (ANOVA) were carried out using Statistical Analysis System software (SAS Institute, Cary, NC, USA).

### 3.5. HPLC Analysis

St. John’s wort leaf extracts were injected into an HPLC (Hitachi L-7400, Tokyo, Japan) using a Mightysil RP-18 GP column (250 mm × 4.6 mm, Kanto Chemical, Tokyo, Japan) for the analysis of hyperforin, rutin, and melatonin.

For the hyperforin assay, the extraction solution was eluted by a mixture of 0.3% phosphoric acid in water and acetonitrile, at a ratio of 10:90 and a flow rate of 1.5 mL min^−1^, the column oven was set at 30 °C, and the UV detector was set at a wavelength of 273 nm.

For the rutin assay, mobile phase A (acetonitrile/water/*o*-phosphoric acid at a ratio of 19:80:1) and mobile phase B (acetonitrile/methanol/*o*-phosphoric acid at a ratio of 59:40:1) were used for gradient elution: 90% mobile phase A to 74% for the first 5 min, before decreasing to 49% between 5 and 10 min, and then decreasing to 0% for the last 5 min. The flow rate was set at 1.0 mL min^−1^, the column oven was set at 25 °C, and the UV detector was set at a wavelength of 284 nm.

For the melatonin assay, the extraction solution was eluted by a mixture containing 82% water, 1.5% phosphoric acid, and 16.5% acetonitrile. The flow rate was set at 1.0 mL min^−1^, the column oven was set at 40 °C, and the UV detector was set at a wavelength of 280 nm. Calibration curves were prepared from hyperforin, rutin, and melatonin standards, and samples were analyzed by comparing retention times with those of the standards.

## 4. Conclusions

Models of cultivation conditions for the accumulation of hyperforin and rutin in St. John’s wort were successfully developed. The optimal conditions for obtaining maximum content of hyperforin (5.6 mg/g) were: temperature of 19 °C, nutrient solution concentration of 45%, and light intensity of 49 μmol m^−2^ s^−1^ PPFD. The optimal conditions for obtaining maximum content of rutin (3.8 mg/g) were: temperature of 18 °C, nutrient solution concentration of 50%, and light intensity of 147 μmol m^−2^ s^−1^ PPFD. We also described RSM models for estimating the content of hyperforin, rutin, and melatonin in the leaves of St. John’s wort affected by different levels of light intensity, temperature, and nutrient concentration. The production yield of leaf biomass increased with light intensity, temperature, and nutrients. The maximum production yield of hyperforin was 5.19 mg/plant at temperature of 23 °C, nutrient solution concentration of 50%, and light intensity of 98 μmol m^−2^ s^−1^ PPFD. The maximum production yield of 3.76 mg/plant was obtained at a temperature of 28 °C, a nutrient solution concentration of 75%, and a light intensity of 147 μmol m^−2^ s^−1^ PPFD. RSM studies on the effect of cultivation variables on the content, and production yield, of hyperforin and rutin will further facilitate the economics of St. John’s wort when scaling up production at a plant factory.

## Figures and Tables

**Table 1:** Box-Behnken design in coded and actual levels of variables and the response functions.

Run	Independent Variable			Responses		
	Temp X1 (°C)	Light Intensity X2 (μmol m^−2^ s^−1^ PPFD)	Nutrient X3 (%)	Hyperforin Content ^b^ (Y_1_) (mg/g)	Rutin Content (Y_2_) (mg/g)	Melatonin Content (Y_3_) (μg/g)
1	−1 ^a^ (18)	−1 (49)	0 (50)	5.653 ± 0.106	0.825 ± 0.043	0.046 ± 0.040
2	−1 (18)	0 (98)	−1 (25)	3.314 ± 0.062	2.138 ± 0.038	0.048 ± 0.038
3	−1 (18)	0 (98)	1 (75)	2.891 ± 0.072	2.474 ± 0.005	0.060 ± 0.048
4	−1 (18)	1 (147)	0 (50)	2.865 ± 0.059	4.107 ± 0.020	0.048 ± 0.039
5	0 (23)	−1 (49)	−1 (25)	4.328 ± 0.051	0.622 ± 0.024	0.059 ± 0.042
6	0 (23)	−1 (49)	1 (75)	3.658 ± 0.074	0.537 ± 0.036	0.049 ± 0.039
7	0 (23)	0 (98)	0 (50)	4.840 ± 0.080	2.340 ± 0.039	0.048 ± 0.039
8	0 (23)	0 (98)	0 (50)	4.920 ± 0.102	2.380 ± 0.050	0.052 ± 0.050
9	0 (23)	0 (98)	0 (50)	4.760 ± 0.055	2.300 ± 0.042	0.044 ± 0.043
10	0 (23)	1 (147)	−1 (25)	3.528 ± 0.086	2.767 ± 0.041	0.063 ± 0.039
11	0 (23)	1 (147)	1 (75)	2.656 ± 0.066	2.565 ± 0.031	0.073 ± 0.039
12	1 (28)	−1 (49)	0 (50)	3.667 ± 0.072	1.379 ± 0.030	0.057 ± 0.040
13	1 (28)	0 (98)	−1 (25)	2.636 ± 0.057	2.565 ± 0.032	0.054 ± 0.039
14	1 (28)	0(98)	1 (75)	2.990 ± 0.081	2.832 ± 0.021	0.058 ± 0.039
15	1 (28)	1 (147)	0 (50)	4.088 ± 0.061	3.109 ± 0.023	0.052 ± 0.040

^a^ The values −1, 0, and 1 are coded levels; ^b^ the content is defined as the weight of the compound per the weight of the leaf.

**Table 2:** ANOVA for response surface models pertaining to the content of bioactive compounds.

Source	Hyperforin		Rutin		Melatonin	
	Sum of Squares	*p*-Value	Sum of Squares	*p*-Value	Sum of Squares	*p*-Value
Linear	2.722	0.0074 ^a^	10.572	0.0004 ^a^	0.0001	0.3715 ^b^
Quadratic	6.63	0.0010 ^a^	1.852	0.0227 ^a^	0.0003	0.1387 ^b^
Crossproduct	2.735	0.0073 ^a^	0.606	0.1588 ^b^	0.0001	0.4395 ^b^
Total Model	12.090	0.0020 ^a^	13.032	0.0023 ^a^	0.0006	0.2751 ^b^
	R^2^ = 0.9736		R^2^ = 0.9717		R^2^ = 0.7608	

^a^ Significant at *p*-value less than 0.05; ^b^ Insignificant at *p*-value more than 0.05.

**Table 3:** Analysis of variance for joint test.

Factor	Hyperforin		Rutin	
	Sum of Squares	*p*-Value	Sum of Squares	*p*-Value
Temperature (x_1_)	4.6490	0.0037 ^a^	1.3564	0.0658
Light intensity (x_2_)	4.7894	0.0035 ^a^	11.8409	0.0006 ^a^
Nutrient concentration (x_3_)	5.8379	0.0022 ^a^	0.3170	0.4680

^a^ Significant at *p*-value less than 0.05.

**Table 4:** Box-Behnken design in coded and actual levels of variables and the response functions.

Run	Independent Variable			Responses (Production Yield ^b^)		
	Temp X1 (°C)	Light Intensity X2 (μmol m^−2^ s^−1^ PPFD)	Nutrient X3 (%)	Total Weight of Leaves (Y_4_) (g/plant)	Hyperforin (Y_5_) (mg/plant)	Rutin (Y_6_) (mg/plant)
1	−1^a^ (18)	−1 (49)	0 (50)	0.709 ± 0.130	4.007 ± 0.734	0.584 ± 0.107
2	−1 (18)	0 (98)	−1 (25)	0.762 ± 0.098	2.525 ± 0.324	1.629 ± 0.209
3	−1 (18)	0 (98)	1 (75)	0.860 ± 0.095	2.486 ± 0.274	2.127 ± 0.235
4	−1 (18)	1 (147)	0 (50)	0.665 ± 0.055	1.905 ± 0.157	2.731 ± 0.225
5	0 (23)	−1 (49)	−1 (25)	0.531 ± 0.176	2.298 ± 0.762	0.330 ± 0.109
6	0 (23)	−1 (49)	1 (75)	0.827 ± 0.228	3.025 ± 0.834	0.444 ± 0.122
7	0 (23)	0 (98)	0 (50)	1.160 ± 0.167	5.614 ± 0.808	2.714 ± 0.390
8	0 (23)	0 (98)	0 (50)	1.046 ± 0.196	5.146 ± 0.964	2.489 ± 0.466
9	0 (23)	0 (98)	0 (50)	1.010 ± 0.061	4.807 ± 0.290	2.323 ± 0.140
10	0 (23)	1 (147)	−1 (25)	0.535 ± 0.059	1.887 ± 0.208	1.480 ± 0.163
11	0 (23)	1 (147)	1 (75)	1.201 ± 0.122	3.189 ± 0.324	3.080 ± 0.312
12	1 (28)	−1 (49)	0 (50)	0.919 ± 0.121	3.369 ± 0.443	1.267 ± 0.166
13	1 (28)	0 (98)	−1 (25)	0.561 ± 0.121	1.478 ± 0.318	1.438 ± 0.310
14	1 (28)	0(98)	1 (75)	1.102 ± 0.135	3.294 ± 0.403	3.120 ± 0.382
15	1 (28)	1 (147)	0 (50)	1.033 ± 0.099	4.222 ± 0.404	3.211 ± 0.307

^a^ The values −1, 0, and 1 are coded levels; ^b^ the production yield is defined as the weight of the leaves, hyperforin, or rutin per plant.

**Table 5:** Independent variables and their levels in coded and actual values.

Independent Variable	Symbol	Coded Variable Levels		
		−1	0	1
Temperature (°C)	X1	18	23	28
Light intensity (μmol m^−2^ s^−1^ PPFD)	X2	49	98	147
Nutrient (%)	X3	25	50	75
